# The Rejection Sensitivity Model: Sexual Minority Adolescents in Context

**DOI:** 10.1007/s10508-019-01572-2

**Published:** 2019-10-29

**Authors:** Laura Baams, Wouter J. Kiekens, Jessica N. Fish

**Affiliations:** 1 Department of Pedagogy and Educational Sciences, University of Groningen, Grote Rozenstraat 38, 9712 TJ Groningen, The Netherlands; 2 Department of Sociology/Interuniversity Center for Social Science Theory and Methodology, University of Groningen, Groningen, The Netherlands; 3 Department of Family Science, School of Public Health, University of Maryland, College Park, MD USA

## Introduction

Theoretical and empirical integration of the rejection sensitivity (RS) model to sexual minority people is one of the few attempts to extend existing theoretical frameworks that explain mental health disparities for this population, namely the minority stress framework (Meyer, 2003) and its extensions (Hatzenbuehler, 2009; Testa, Habarth, Peta, Balsam, & Bockting, 2015). Theoretical origins of RS are rooted in the desire to understand how rejection from significant others affects subsequent other close relationships (Downey & Feldman, 1996). This was later extended to conceptualize rejection based on membership of a stigmatized group and modified to understand sexual orientation-related RS among sexual minorities (Dyar, Feinstein, Eaton, & London, 2016; Pachankis, Goldfried, & Ramrattan, 2008). Feinstein (2019) brings new life to this adapted application by grounding and integrating the basic tenets of sexual orientation-related RS alongside a critical health compromising process of minority stress: vigilance. Meyer theorized vigilance as a core form of proximal minority stressors and explains that “LGB people learn to anticipate—indeed, expect—negative regard from members of the dominant culture. To ward off potential negative regard, discrimination, and violence, they must be vigilant” and this vigilance is “related to feared possible (even if imagined) negative events” (Meyer, 2003, p. 680–681). Feinstein explains that existing theoretical frameworks (Hatzenbuehler, 2009; Meyer, 2003) mention vigilance and RS as important processes, but lack a comprehensive integration of these concepts. Given that schemas for RS are formed early in the life course, we focus on the applicability to sexual minority adolescents, and other marginalized groups.

## How Rejection Sensitivity Might Expand Understandings of LGBTQ Adolescent Mental Health

Overwhelmingly, research testing the negative impacts of minority stress among sexual minority adolescents has focused on mental health, and more specifically depression and suicidality. With good reason, studies show that sexual minority adolescents experience depression (Lucassen, Stasiak, Samra, Frampton, & Merry, 2017) and suicidality (Salway et al., 2019) at much higher rates than heterosexual adolescents, sometimes as young as age 11 (La Roi, Kretschmer, Dijkstra, Veenstra, & Oldehinkel, 2016). A smaller subsection of this research has enumerated anxiety as a correlate of minority stress (Jones, Robinson, Oginni, Rahman, & Rimes, 2017). The RS framework presented by Feinstein (2019) includes “anxiety” as an important feature of RS: To “anxiously expect rejection” is distinct from expecting rejection in the absence of anxiety or fear. This is a crucial distinction, because it is the anxiety that accompanies these expectations which activate RS as a detrimental process for sexual minority adolescents’ mental health.

The integration of the RS framework may also increase understanding of other important consequences of differential anticipatory emotions of rejection, namely anger and aggression. We may, for example, see different associations between RS and externalizing or internalizing behaviors as a result of anticipatory responses that reflect anger relative to anxiety. In a study among lesbian and bisexual women, for example, results showed that drinking expectancies of aggression and anger were associated with alcohol abuse and dependency, whereas expecting that drinking will help you forget your worries when depressed was associated with heavy episodic drinking (Fish & Hughes, 2018). Research investigating victimization and bullying (Lereya, Copeland, Zammit, & Wolke, 2015) may benefit from testing the role of different anticipatory emotions as these might influence outcomes of aggression (anticipation of anger) relative to poor mental health (anticipation of anxiety). Aligning with Feinstein’s (2019) framework, we might hypothesize that some adolescents who are rejected may be more likely to respond with aggression. For example, a study among Minnesotan adolescents showed that sexual minority adolescents were more likely to be both victims and perpetrators of bullying behaviors than their heterosexual peers (Eisenberg, Gower, McMorris, & Bucchianeri, 2015). At the same time, there could be positive, adaptive features of anticipation of anger in response to rejection: Shared anger may bring about and strengthen community, activism, civic engagement, and other forms of resilience among sexual minority adolescents through, for example, Gender and Sexuality Alliances in schools (Poteat, Scheer, Marx, Calzo, & Yoshikawa, 2015). Youth may also anticipate and cope with negative experiences by hyper-engaging in school (Watson & Russell, 2016). Whether anxious or angry expectations of rejection bring about mental and behavioral problems, or positive change, likely depends on the context in which the rejection takes place and the support sexual minority youth might find in other contexts. Importantly, the distinction between these anticipatory emotions in relation to externalizing and internalizing behaviors may also require different intervention strategies (e.g., targeting anger versus targeting youth anxiety).

## Critical Developmental Periods for Rejection Sensitivity

Research shows that sexual orientation disparities in victimization are evident at young ages, well before many adolescents acknowledge an awareness of their sexual orientation (Martin-Storey & Fish, 2019; Mittleman, 2019). Might these early experiences of rejection become part of the learning history that sets an early precedent for elevated sexual orientation-specific RS across the life course? Further, considering the declining age of coming out for adolescents, experiences with rejection and internalization of negative messaging around sexual minority identity may come at a younger age than previous generations, when both mental health (Russell & Fish, 2019) and RS are more vulnerable to these unique social stressors.

According to Feinstein (2019), the cognitive social learning history of sexual minority individuals might also include vicarious experiences of rejection that impact their own RS (e.g., witnessing victimization or hearing negative messages about sexual minority individuals in media). Although media has become more inclusive of sexual (and gender) diversity, debates around marriage equality, bathroom access, and conversion therapy might also portray negative views of sexual and gender diversity. Thus, in addition to witnessing hate crimes, (social) media portrayals of violence against sexual minority individuals might impact young people’s anxious expectations of the world (Paterson, Brown, & Walters, 2019). A parallel process can be observed in children and youth of color, who experience vicarious racism negatively impacting various health outcomes (Heard-Garris, Cale, Camaj, Hamati, & Dominguez, 2018). Importantly, vicarious experiences may be more or less impactful depending on when they occur in the life course, in that contemporary cohorts of sexual minority youth may be uniquely influenced by the negative political rhetoric in ways that alter their RS and hypervigilance across the life course. In fact, some might argue that the degree to which contemporary youth engage in new media may make this cohort of sexual minority youth particularly susceptible to RS, and the negative mental health consequences therein.

With rapid changes in social attitudes toward sexual and gender diversity, research has increasingly included a focus on cohort differences among sexual minority people. Our focus on adolescence is in part due to the developmental stage and its importance for the development of RS, but also because contemporary sexual minority adolescents grow up in a social environment that is unique from older cohorts of sexual minority adults—and its impact on their lived experience and health will likely play out in unforetold ways across their life course. For example, in a study examining experiences of discrimination across the life course, gay and bisexual men in their 40s and 50s evidenced the highest rates of perceived discrimination relative to lesbian, gay, and bisexual adults of other ages (both older and younger). Rice, Fish, Russell, and Lanza (2019) attributed this anomoly to the fact that these men would have been in their late teens and early 20s during the AIDS epidemic, therefore (potentially) heightening these men’s perceptions of discrimination. We imagine, given the tenets of RS, that these men might have altered attributions of potentially neutral or ambiguous stimuli and elevated RS given the social stigma they experienced during this contentious time in history. Growing attention for cohort differences in perceptions of discrimination and compromised health might foreshadow how different cohorts have unique RS-inducing experiences, and therefore unique health consequences.

## Sociocontextual Perspectives on Rejection Sensitivity Processes

Existing theoretical frameworks, including the current RS framework, pay little attention to the context in which people develop RS or experience early rejection. As Feinstein (2019) outlines, context matters both for the occurrence and salience of rejection and may impact the development of RS. In addition, experiencing rejection in spaces that are thought to be “safe” might have a stronger impact on RS than experiencing rejection in historically unaccepting spaces. For example, when bisexual individuals experience rejection in the LGBTQ community, this may impact their RS to a larger extent than when they experience rejection in historically heterosexual spaces (Bostwick & Hequembourg, 2014). Additionally, experiencing RS in “safe” spaces might elicit different anticipatory emotions (e.g., anxiety, anger) compared to experiencing RS in more traditionally unaccepting spaces. Further, many sexual minority adolescents experience victimization in multiple contexts: their family home, school, or in public (Goldbach & Gibbs, 2017). Experiences in one context might translate to elevated RS in other contexts. For RS in particular, it is important to attend to multiple intersecting contexts and how experiences with rejection in one context does not preclude the experience of rejection in another, and may even set in motion the development of “generalized” RS, because it likely manifests as the result of experiences across multiple contexts. Alternatively, sexual minority adolescents might also gradually select more supportive social environments (i.e., friends, LGBTQ community) limiting experiences with rejection and increasing positive and supportive experiences (Cohen, Padilla, & Aravena, 2006).

## Applicability to Other Marginalized/Minority Groups

Preliminary research on RS supports the notion that processes of RS explain the associations between minority stress and health outcomes, and that this mechanistic process holds for different sexual minority subgroups (e.g., men/women, monosexual/bisexual sexual minority people; Feinstein, 2019), which is what one hopes to see when theory building. However, Feinstein reflects on how anxious expectations of rejection might differ when we account for intersections with gender, race/ethnicity, and age, and that cues triggering anxious expectations may be different from cues triggering angry expectations among specific groups.

A focus on intersecting experiences of rejection is crucial in understanding the development of RS for many sexual minority youth. That is, sexual minority youth of color likely develops RS related to two social statuses: sexual orientation-related RS from society in general, as well as race/ethnicity-related RS from both society in general, but also within the LGBTQ community, whereas White sexual minority people likely do not experience RS related to racial/ethnic identity. Along these same lines, however, RS related to different identities—such as a racial/ethnic identity—may also cultivate unique strategies to combat the negative influence of rejection, staving off RS. For example, parents of youth of color often engage in conversations around race-related experiences, including race-related rejection (i.e., family racial/ethnic socialization practices), which has been shown to have positive effects for mental health and academic achievement among youth of color (Hughes et al., 2006). It could be that these strategies to address race-related rejection could help sexual minority youth of color cope with sexual orientation-related rejection. Thus, intersectional views on the development of RS could provide new perspectives on “multiple minority stressors” and how these experiences are linked to resilience, but also compromised mental health.

In addition, although Feinstein (2019) acknowledges the applicability of the RS framework to different genders, there is currently very little research on the development of RS among transgender and other gender minority adolescents. Theoretical work on gender minority stress mentions negative expectations from the future as an important precursor of vigilance (Rood et al., 2016; Testa et al., 2015) and suggests that experiences with rejection and trauma in transgender persons form a “trauma history” which then creates vigilance similar to how it would in sexual minority populations (Hendricks & Testa, 2012). However, because we currently lack a measure of RS that is applicable to multiple groups and groups with multiple minority identities, we have very little knowledge of the experience and development of RS among gender minority adolescents. Further, the emergence of new identity labels among sexual and gender minority youth (e.g., pansexual, genderqueer, gender nonbinary) underline the need for measurement development for a diverse and dynamic sexual and gender minority population (Galupo, Ramirez, & Pulice-Farrow, 2017).

## Intervention versus Prevention

Feinstein (2019) broadly discusses currently available interventions that target RS, such as cognitive behavioral therapy (CBT). An alternative to CBT may be to target the memory bias—an important aspect of RS—through trauma-informed care or eye movement desensitization and reprocessing (EMDR) therapies (Pantalone, Valentine, & Shipherd, 2017). However, a commonality among these psychological approaches is that they rely on people finding their way to, and accessing, mental health providers. Adolescents with high levels of RS might struggle to do so, may not be out to parents, and might prefer not to disclose their sexual orientation to health providers (Fuzzell, Fedesco, Alexander, Fortenberry, & Shields, 2016). Similarly, although people may suffer from mental health and substance use disorders, relatively few people engage in formal counseling or therapy (Lipari, Park-Lee, & Van Horn, 2016; SAMHSA, 2016). It is therefore necessary to understand how large-scale intervention strategies might be developed or adapted to address and intervene in RS processes for sexual minority youth.

Further, Feinstein (2019) and the RS literature, more broadly, do not pay much attention to how RS might be prevented. For example, rejection might occur in one context (e.g., school) but if other contexts are supportive (e.g., home), the development of RS might be slowed down or prevented. For sexual minority adolescents, the school context might be particularly amenable to change by implementing inclusive policies and programs that reduce bullying and improve acceptance (Day, Ioverno, & Russell, 2019). LGBTQ community centers (see Fish, Moody, Grossman, & Russell, 2019; Williams, Levine, & Fish, 2019) may also be uniquely positioned to deliver programs that increase sexual minority youth’ self-esteem and develop coping strategies to address RS and the associated mental health consequences. Additionally, psychoeducation and advocacy has improved support and acceptance in the family context (Parker, Hirsch, Philbin, & Parker, 2018), while medical systems might benefit from education and training to improve accessibility and cultural competence (Bidell & Stepleman, 2017).

## Conclusion

In addition to the applicability of the RS framework for sexual minority individuals from various cohorts, the applicability of this framework to different sexual and gender minority populations remains to be seen. Research among diverse groups of sexual and gender minority individuals should consider important aspects of identity development, such as identity centrality (Dyar et al., 2016). Although identity development clearly plays a role in the development of RS, these mechanisms are likely universal, whereas the trigger of RS is minority (or status)-specific. What Feinstein’s (2019) visioning does is portray a close-up of one aspect of the minority stress framework, which enables us to more rigorously test hypotheses and improve the specificity of the minority stress framework. By doing so, Feinstein offers an example of what scholars can do to continue to innovate minority stress theory and thereby improve our understanding of how minority stress impacts health and the ways we can disrupt this process.
